# A Novel Sea Anemone Peptide Ed-79 Blocks Shaker Kv Channels and Exhibits Insecticidal Activity

**DOI:** 10.3390/molecules31213822

**Published:** 2026-10-23

**Authors:** Mengxin Yue, Yanling Liao, Tianru Hu, Qin Chen, Xuefeidan Liu, Bingmiao Gao, Qiqi Guo

**Affiliations:** 1 Hainan Key Laboratory for Research and Development of Tropical Herbs, Engineering Research Center of Tropical Medicine Innovation and Transformation of Ministry of Education, College of Pharmacy, Hainan Medical University, Haikou 571199, China; 2 School of Agricultural and Rural Affairs, Hainan Open University, Haikou 570028, China; 3 Department of Pharmacy, Cangnan Hospital of Wenzhou Medical University, Wenzhou 325800, China

**Keywords:** sea anemone venom, insecticidal activity, Shaker Kv channel, molecular docking

## Abstract

The escalating resistance of agricultural pests to conventional chemical insecticides has created an urgent need for eco-friendly biological alternatives with novel modes of action. Sea anemone venoms are rich in structurally stable and target-specific ion channel-modulating peptides, representing a promising source of new insecticidal molecules. Through bioinformatics screening of the transcriptome database of the sea anemone *Exaiptasia diaphana*, we identified a novel 37-residue peptide, Ed-79, which contains three disulfide bonds. The peptide was chemically synthesized via solid-phase peptide synthesis (SPPS) and successfully folded into its correct disulfide-bonded form using a three-step oxidative folding strategy, as confirmed by LC-MS and RP-HPLC. Circular dichroism (CD) spectroscopy revealed marked secondary-structural changes upon oxidative folding, notably an increased β-sheet content. In insecticidal bioassays, Ed-79 exhibited dose-dependent lethality against *Spodoptera frugiperda* and *Tenebrio molitor*, with LD_50_ values of 289.5 and 367.2 pmol/g, respectively. Molecular docking further predicted that Ed-79 binds stably to the outer vestibule of the Shaker Kv channel pore via eight hydrogen bonds, with Pro475 and Asn482 potentially serving as key interacting residues. Collectively, this is the first systematic report on the synthesis, oxidative folding, insecticidal activity, and predicted Shaker Kv channel-binding mode of Ed-79, providing a solid foundation for developing new insecticidal lead compounds and broadening the utility of anemone venoms as insecticide resources.

## 1. Introduction

The escalating resistance of agricultural pests to traditional chemical pesticides has become a critical threat to global food security and ecological sustainability [1,2,3,4]. The prolonged and widespread use of conventional insecticides, including pyrethroids, organophosphates, and neonicotinoids, has driven numerous mutations in target sites (e.g., voltage-gated sodium channels and acetylcholinesterase), substantially reducing pest control efficacy [5,6]. Consequently, the discovery of naturally occurring bioactive molecules with novel mechanisms of action, particularly neuroactive peptides derived from animal venoms [7,8], has emerged as a key strategy for developing next-generation green biocides [9].

The sea anemone *Exaiptasia diaphana*, a member of one of the oldest cnidarian lineages, produces venom rich in structurally stable, target-specific ion-channel-modulating peptides in its stinging cells [10]. Among these, potassium channels, especially voltage-gated K^+^ channels [11] of the Shaker family, have become attractive targets for insecticidal peptides [12], owing to their central roles in regulating neuronal excitability [13], neurotransmitter release, and muscle contraction in insects. To date, several anemone-derived K^+^ channel inhibitors (e.g., ShK and BDS) have been shown to bind mammalian Kv1.x channels with high affinity [14,15,16,17,18], and some of these peptides have also been reported to block insect Shaker voltage-gated potassium channels. However, compared with the extensive research on scorpion and spider venoms [19,20], studies on insecticidal peptides from sea anemones remain relatively limited, particularly in terms of systematic workflows that integrate omics-based discovery, synthetic optimization, and functional validation.

In this study, the *E. diaphana* transcriptome database previously established in our laboratory [21] was employed as a starting point for peptide discovery. Following bioinformatics screening and molecular docking analysis [22], a novel 37-residue peptide containing three disulfide bonds was identified and designated Ed-79.

Structural predictions and homology searches suggested that Ed-79 may bind to voltage-gated K^+^ channels [23], although this interaction has not yet been experimentally validated. To further validate this hypothesis, we performed molecular docking using the high-resolution structure of the *Drosophila melanogaster* Shaker Kv channel in its open-conducting state (PDB ID: 7SIP) [24] which shares a highly conserved pore region and selectivity filter with Shaker Kv channels of agricultural pests. Shaker orthologs are highly conserved among insects, and the selectivity filter signature sequence TMTTVGYG is identical across *Lepidoptera* and *Coleoptera*. The results indicated that Ed-79 binds stably to the outer vestibule of the channel pore, resembling the mode of action of known K^+^ channel blockers [25]. More importantly, BLASTp searches identified six homologous peptides that have been previously reported to block Shaker Kv channels or exhibit insecticidal activity against various agricultural pests. Multiple sequence alignment [26] revealed that these homologs share a conserved structural framework and key functional residues, raising the possibility that Ed-79 may also possess insecticidal properties. However, this hypothesis requires experimental validation.

Against this background, the present study aims to systematically investigate the insecticidal activity of Ed-79 and its predicted binding mode with the Shaker Kv channel [27]. Our specific objectives are (1) the chemical synthesis and oxidative folding of Ed-79 [28]; (2) in vivo injection bioassays and insecticidal activity assays against *Spodoptera frugiperda* and *Tenebrio molitor*; (3) molecular docking to predict its binding to the Shaker Kv channel; and (4) sequence and structural comparison with homologous peptides. This work not only provides a foundation for developing novel insecticidal lead compounds [29] but also broadens our understanding of sea anemone venom as a valuable insecticide resource [30].

## 2. Results

### 2.1. Homology Alignment and 3D Structural Prediction of the Sea Anemone Peptide Ed-79

To discover novel venom peptides from *E. diaphana*, six previously reported ShK-family peptides known to block Shaker Kv channels or exhibit insecticidal activity against agricultural pests were first selected from the literature. These sequences served as queries for homology-based alignment and screening against the *E. diaphana* transcriptome database previously established in our laboratory. This screening led to the identification of a novel 37-residue peptide, designated Ed-79, which contains six cysteine residues predicted to form three disulfide bonds.

**Figure 1:**
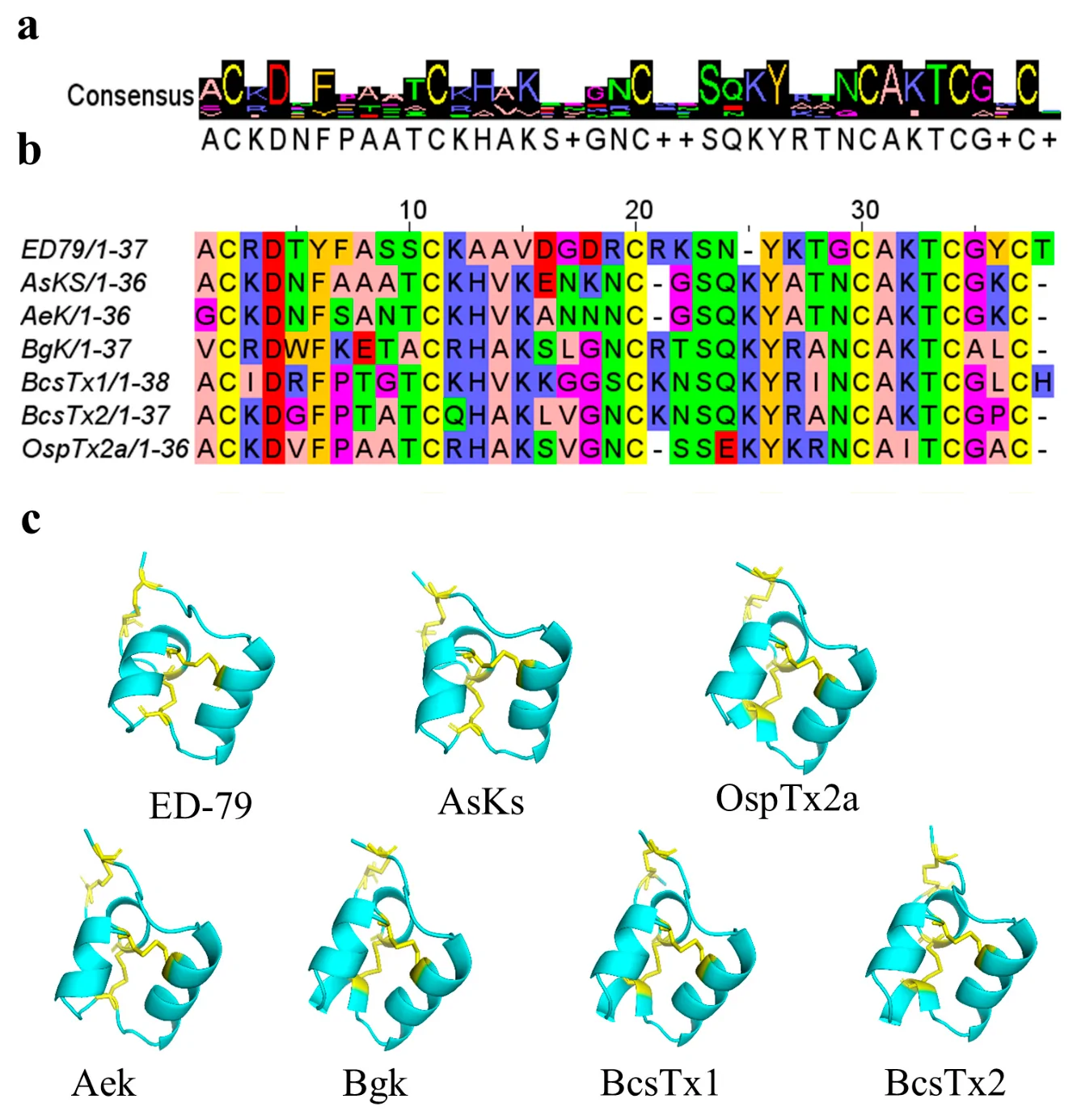
Sequence and structural characterization of peptide Ed-79. (**a**) WebLogo depicting conserved sequence motifs shared between Ed-79 and its homologs. (**b**) Multiple sequence alignment of Ed-79 with six homologous sea anemone peptides: AsKs (UniProt: Q9TWG1), OspTx2a (UniProt: A0A330KW27), AeK (UniProt: P81897), BgK (UniProt: P29186), BcsTx1 (UniProt: C0HJC2), and BcsTx2 (UniProt: C0HJC3). Strictly conserved cysteine residues are boxed in yellow. (**c**) AlphaFold-predicted three-dimensional structure of Ed-79, showing disulfide bonds as yellow sticks.

WebLogo analysis of the conserved sequence motifs shared between Ed-79 and its homologs revealed a consensus pattern characterized by a strictly conserved cysteine framework (Figure 1a), consistent with the ShK-family fold. Multiple sequence alignment using Jalview software (Figure 1b) shows that Ed-79 shares a highly conserved cysteine backbone with these known ShK-family peptides. The six cysteine residues are conserved and are predicted to form three pairs of disulfide bonds with the connectivity C1–C6, C2–C4, and C3–C5. In the alignment, all cysteine positions are strictly conserved (highlighted in yellow in Figure 1b), indicating that Ed-79 retains the typical ShK-fold cysteine framework. Furthermore, in the putative functional regions—particularly the loop region spanning residues 20–29, which corresponds to the Shaker Kv channel-binding surface—the key functional residues in Ed-79 correspond to the functionally important dyad in ShK-family channel-blocking peptides (Figure 1b), suggesting that it could potentially share a similar channel-binding mode. Three-dimensional structure prediction using AlphaFoldv3.0 revealed that Ed-79 and its six homologs adopt a conserved ShK-family fold, characterized by a compact β-sheet core stabilized by three disulfide bonds (Figure 1c). The predicted structures of all seven peptides were highly similar, further supporting the structural conservation within this peptide family.

### 2.2. Chemical Synthesis and Sequential Oxidative Folding of Ed-79

**Figure 2:**
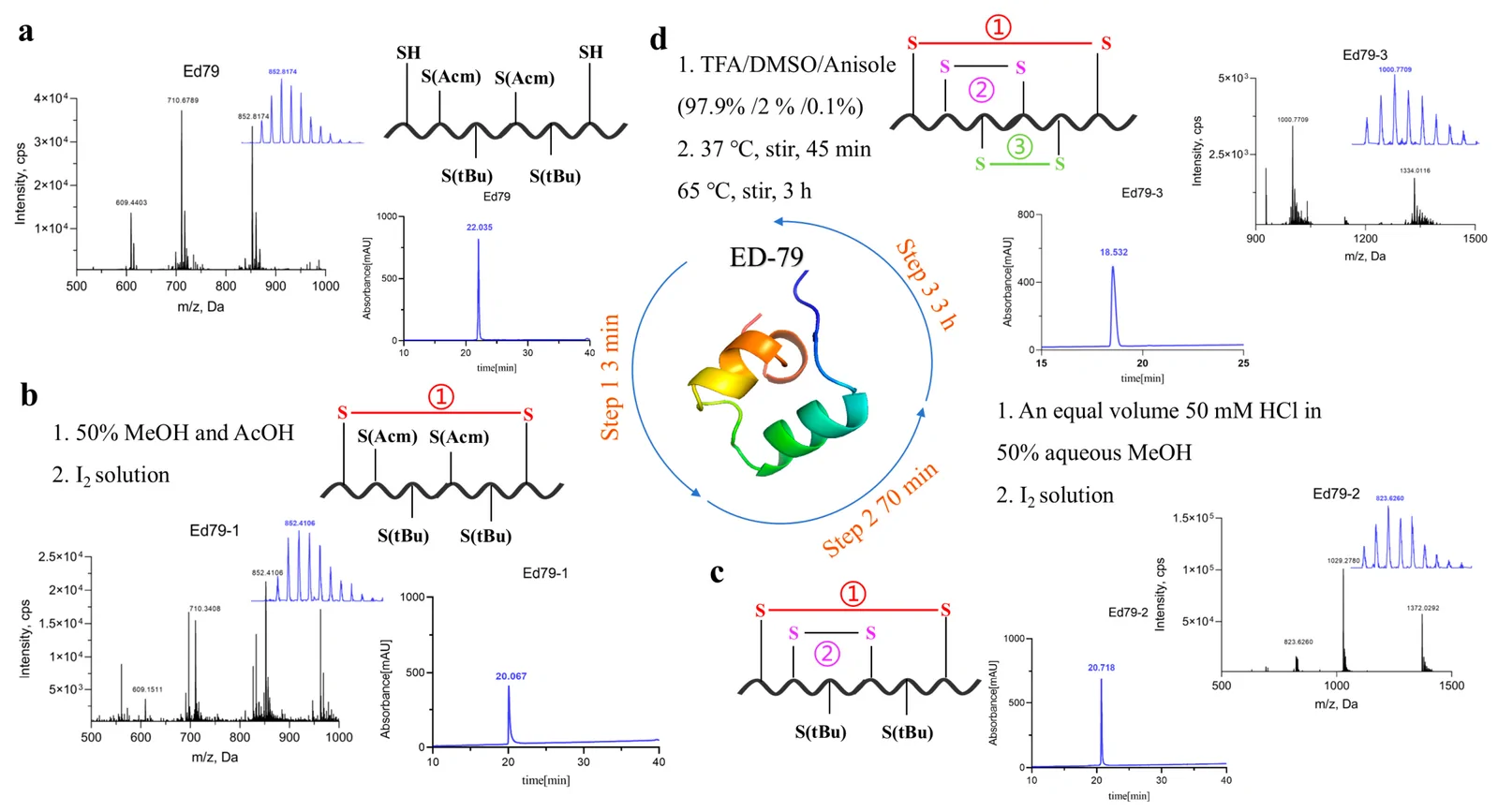
LC-MS and RP-HPLC characterization of the synthetic linear Ed-79 peptide and its sequential oxidative folding intermediates. (**a**) Analytical RP-HPLC chromatogram and LC-MS spectrum of the protected linear precursor. (**b**) After the first oxidation step, the molecular weight decreased and the retention time shifted, consistent with formation of the first disulfide bond. (**c**) After the second oxidation step, the molecular weight further decreased and the retention time shifted, corresponding to removal of two Acm groups and formation of the second disulfide bond. (**d**) After the third oxidation step, the molecular weight decreased further and the retention time decreased, corresponding to removal of two tBu groups and formation of the third disulfide bond.

The protected linear precursor of Ed-79 was synthesized by solid-phase peptide synthesis (SPPS) and purified by preparative reversed-phase high-performance liquid chromatography (RP-HPLC). The precursor contained two free cysteine residues, two Cys(Acm) residues, and two Cys(tBu) residues. The molecular weight of the purified protected linear precursor was 4259.10 Da, in close agreement with the theoretical value. Its analytical RP-HPLC retention time was 22.035 min (Figure 2a), confirming successful synthesis. After the first oxidation step, the molecular weight decreased to 4257.05 Da (a loss of ~2 Da), and the retention time shifted from 22.035 to 20.067 min (Figure 2b), consistent with the formation of the first disulfide bond through dehydrogenation of the two free cysteine residues. After the second oxidation step, the molecular weight decreased to 4113.13 Da (a loss of ~144 Da); the retention time further shifted to 22.513 min (Figure 2c). This corresponded exactly to the removal of two Acm protecting groups and the formation of the second disulfide bond. After the third oxidation step, the final molecular weight was 3999.08 Da, a further decrease of ~114 Da; the retention time decreased markedly to 18.532 min (Figure 2d), corresponding to the removal of two tBu groups and the formation of the third disulfide bond. All observed molecular weights matched the theoretical values, confirming the successful formation of the three correctly paired disulfide bonds. Collectively, the stepwise changes in molecular weight and RP-HPLC retention time were consistent with the expected sequential deprotection and formation of three disulfide bonds, supporting the successful oxidative folding of Ed-79.

### 2.3. Circular Dichroism Spectroscopy Analysis

**Figure 3:**
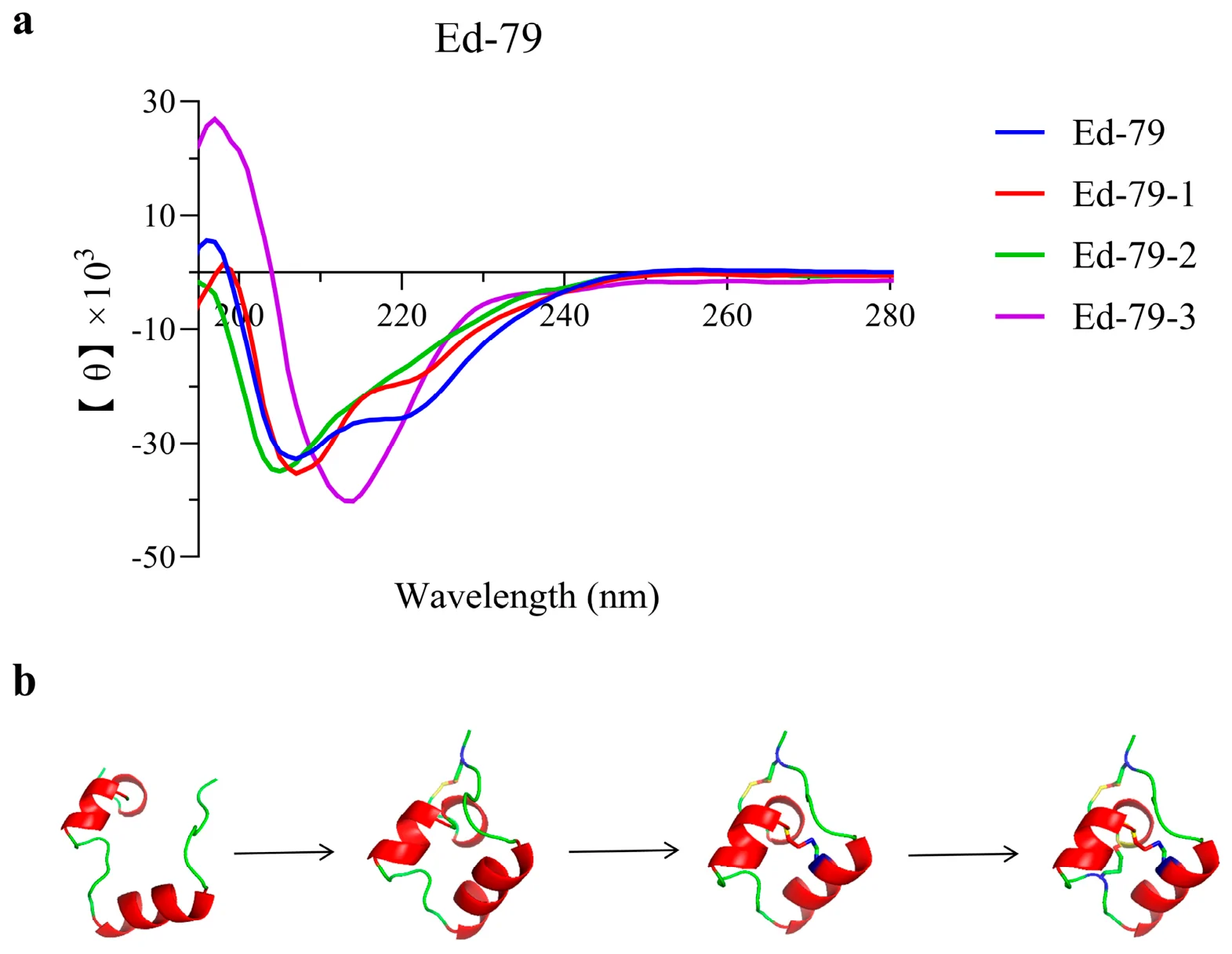
CD spectroscopy and structural models of Ed-79 oxidative folding intermediates. (**a**) CD spectra of linear Ed-79 and its oxidative folding intermediates (Ed-79-1, Ed-79-2, and Ed-79-3) recorded from 190 to 260 nm. (**b**) AlphaFold-predicted structural models illustrating the progressive formation of disulfide bonds, with 0, 1, 2, and 3 disulfide bonds formed from left to right, respectively.

CD spectroscopy was employed to monitor the secondary structural changes of Ed-79 during oxidative folding, and the results are presented in Figure 3. As the three-step oxidation proceeded, the CD spectra exhibited marked changes in both signal shape and intensity, indicating progressive conformational rearrangement of the peptide. Quantitative analysis of the secondary structure components (Table S1) further revealed that, compared with the linear peptide, the fully oxidized Ed-79 showed decreased α-helix content and an increased β-sheet proportion, suggesting that oxidative folding drives the peptide toward a more compact, β-sheet-rich conformation.

### 2.4. Insecticidal Activity of Ed-79 Against Spodoptera frugiperda and Tenebrio molitor

**Figure 4:**
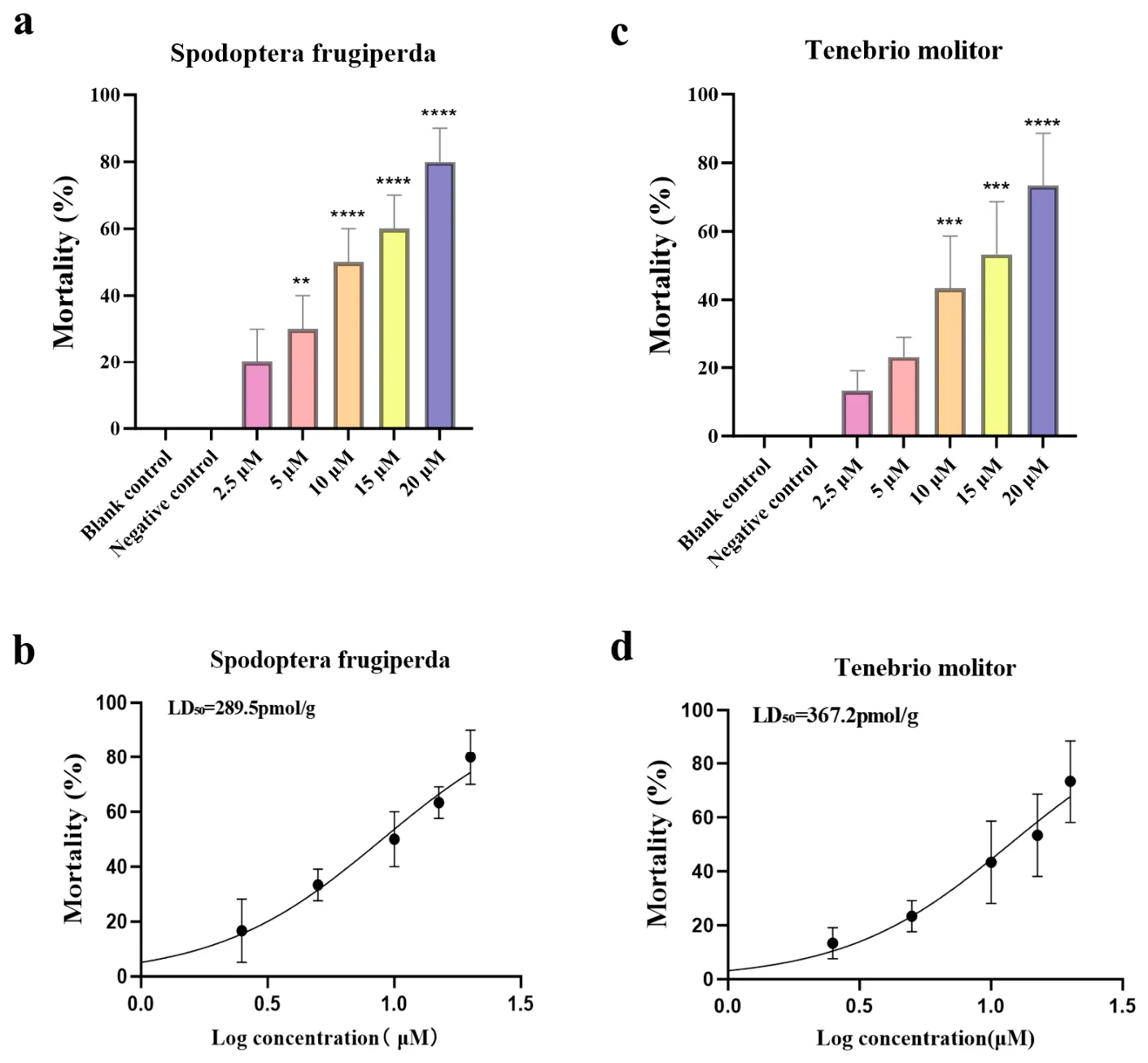
Dose-dependent insecticidal activity of Ed-79 against *S. frugiperda* and *T. molitor*. (**a**) Mortality of *S. frugiperda* larvae at various concentrations of Ed-79. (**b**) Dose–response curve of Ed-79 against *S. frugiperda* with the fitted LD_50_ value. (**c**) Mortality of *T. molitor* larvae at various concentrations of Ed-79. (**d**) Dose–response curve of Ed-79 against *T. molitor* with the fitted LD_50_ value. Data are presented as mean ± SEM (*n* = 10 per group). Asterisks indicate statistically significant differences compared with the negative control group (** *p* < 0.01, *** *p* < 0.001, **** *p* < 0.0001). Dose–response curves were fitted by nonlinear regression using a log(agonist) vs. response model with variable slope in GraphPad Prism v10.1.2.

To evaluate the in vivo insecticidal activity of Ed-79, the synthetic peptide was injected into the lower abdomen of *Spodoptera frugiperda* and *Tenebrio molitor* larvae at concentrations ranging from 2.5 to 20 μM, as shown in Figure 4a,c. Ed-79 exhibited dose-dependent lethal effects on both insect species, whereas the blank and negative control groups showed no observable mortality. At the highest concentration tested (20 μM), Ed-79 caused pronounced mortality in both insects, while no significant effect was detected at the lowest concentration (2.5 μM). The half-lethal doses (LD_50_) of Ed-79 were determined to be 289.5 pmol/g for *S. frugiperda* and 367.2 pmol/g for *T. molitor*.

### 2.5. Molecular Docking of Ed-79 with the Shaker Kv Channel

**Figure 5:**
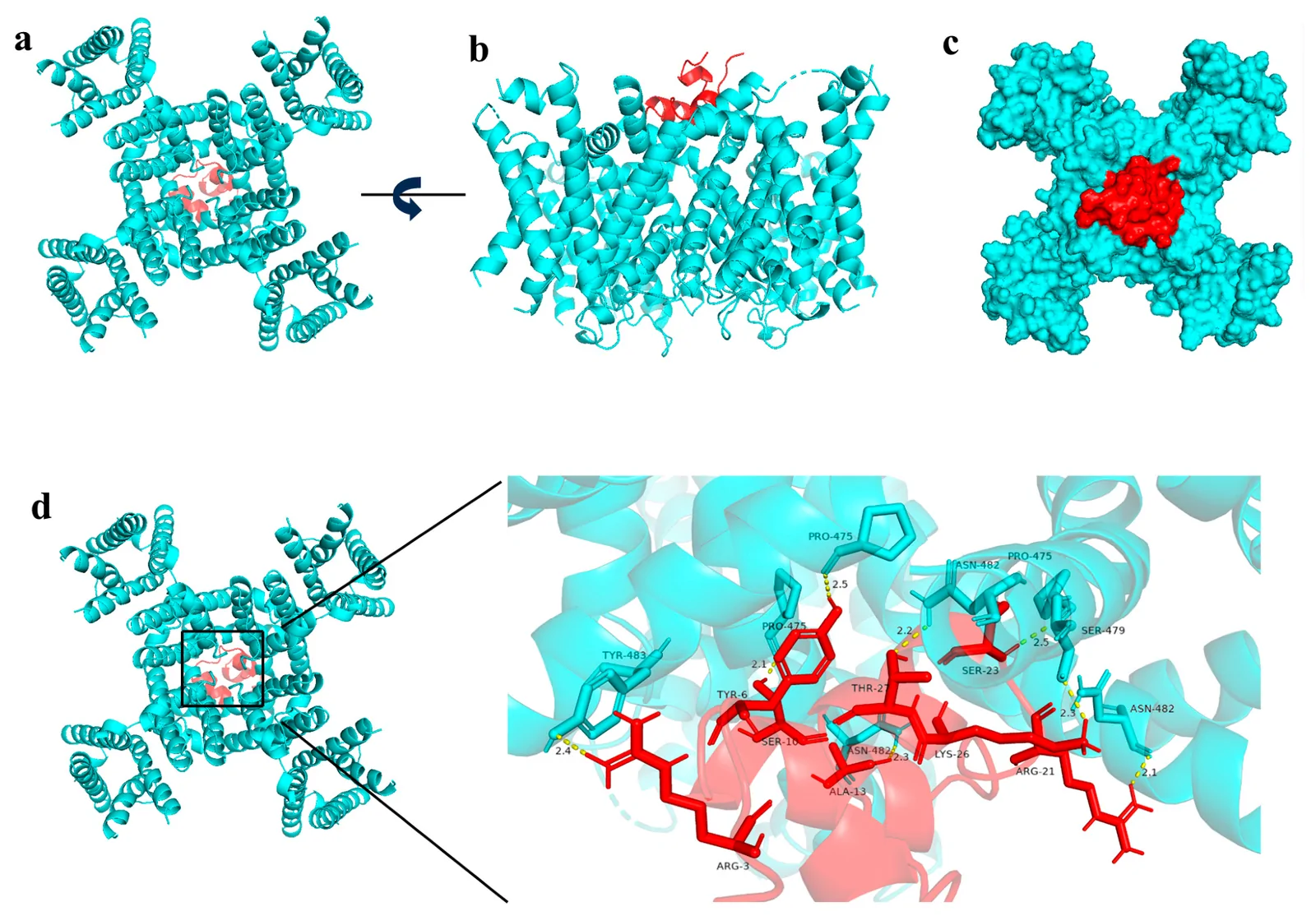
Molecular docking of Ed-79 with the Shaker Kv channel (PDB ID: 7SIP). (**a**) Top view of the docking complex, with Ed-79 shown in red and the Kv channel in cyan. (**b**) Side view of the same complex. (**c**) Close-up view of Ed-79 bound to the channel pore, depicted as a ball-and-stick model. (**d**) Detailed binding interface (boxed region in (**c**)), showing hydrogen-bonding interactions (yellow dashed lines) between Ed-79 and surrounding channel residues. Key interacting residues (Pro475, Asn482, Tyr483, and Ser479) are labeled.

To predict the binding mode of Ed-79 with the Shaker Kv channel (PDB ID: 7SIP), rigid docking (ZDOCK) and energy optimization (RDOCK) were performed using Discovery Studio 2023, and the optimal docking conformation was visualized and analyzed with PyMOL 2.6. As shown in Figure 5, Ed-79 bound stably to the outer vestibule of the channel pore, forming a total of eight hydrogen bonds with surrounding channel residues.

Among these interactions, Pro475 and Asn482 in the channel pore were the most frequently involved residues. Pro475 formed three hydrogen bonds with Ser10, Tyr6, and Ser23 of Ed-79, with distances ranging from 2.1 to 2.5 Å; Asn482 formed three hydrogen bonds with Ala13, Thr27, and Arg21 of the peptide, with distances of 2.1–2.3 Å. In addition, Tyr483 of the channel formed one hydrogen bond with Arg3 (2.4 Å), and Ser479 formed one hydrogen bond with Lys26 (2.3 Å). All hydrogen-bond distances fell within the 2.1–2.5 Å range, characteristic of strong hydrogen-bond interactions.

This extensive hydrogen-bond network, involving multiple polar residues from Ed-79 (Arg, Ser, Thr, Lys, and Tyr), suggests that the peptide specifically recognizes and stabilizes its binding conformation at the pore region of the Shaker Kv channel, with Pro475 and Asn482 potentially serving as key interacting residues. This binding mode is consistent with the known mechanism of pore-blocking potassium channel inhibitors, suggesting that Ed-79 may function as a Shaker Kv channel blocker. However, direct electrophysiological evidence is required to confirm this predicted interaction. The Shaker Kv channel is a tetramer, and Pro475 and Asn482 are present in each of the four subunits. The multiple Pro475 and Asn482 residues shown in Figure 5d correspond to neighboring subunits of the channel that participate in Ed-79 binding.

## 3. Discussion

In this study, we systematically report for the first time the chemical synthesis, oxidative folding, insecticidal activity, and predicted Shaker Kv channel-binding mode of Ed-79, a novel peptide derived from the sea anemone *E. diaphana*. Using SPPS in combination with a three-step oxidative folding strategy [31,32,33], we successfully obtained Ed-79 with the correct disulfide connectivity (C1–C6, C2–C4, and C3–C5), as confirmed by the close match between its observed molecular weight (3999.08 Da) and the theoretical value. Insecticidal bioassays demonstrated that Ed-79 exerts dose-dependent lethal effects against both *S. frugiperda* (fall armyworm) and *T. molitor* (mealworm), with LD_50_ values of 289.5 and 367.2 pmol/g, respectively. Molecular docking further predicted that Ed-79 binds stably to the outer vestibule of the Shaker Kv channel through eight hydrogen bonds (2.1–2.5 Å), with Pro475 and Asn482 potentially serving as key interacting residues.

The insecticidal activity of Ed-79 is closely linked to the specific secondary structure acquired upon oxidative folding. CD spectroscopy [34] revealed that the transition from the linear peptide to the fully oxidized product is accompanied by a marked decrease in α-helix content and a corresponding increase in β-sheet content (Table S1). This structural shift is consistent with the features of known disulfide-rich ion channel-modulating peptides [35,36], such as the anemone toxin ShK and the scorpion toxin charybdotoxin. It is widely recognized that the compact β-sheet framework, stabilized by multiple disulfide bonds, constitutes the structural basis for high-affinity binding of peptide toxins to ion channels [37]. Therefore, the increased β-sheet content observed in Ed-79 may contribute to its bioactive conformation and target recognition.

Ed-79 exhibited dose-dependent lethality against both insect species when injected into the larval hemocoel, with LD_50_ values of 289.5 and 367.2 pmol/g, respectively. These values are within the range reported for insecticidal venom peptides administered by injection [38,39]. However, the insecticidal potency of Ed-79 via other routes of administration (e.g., oral feeding) remains to be determined.

The Shaker Kv channel structure (PDB ID: 7SIP) was derived from *Drosophila melanogaster* and resolved at 3.0 Å resolution using cryo-electron microscopy, representing a high-resolution structure of an insect voltage-gated potassium channel. The Shaker Kv channel is highly conserved among insects, and the selectivity filter signature sequence TMTTVGYG is identical across *Lepidoptera* and *Coleoptera* [40]. Given its essential role in regulating neuronal excitability and muscle contraction, the Shaker Kv channel has been recognized as a promising target for insecticide development. Recent studies have demonstrated that scorpion venom-derived peptides such as LaIT6 exhibit significant insecticidal activity by selectively inhibiting insect K^+^ channels without affecting mammalian channels, further validating the potential of targeting Shaker Kv channels for pest control. These findings support our molecular docking approach to predict the binding mode of Ed-79 to the Shaker Kv channel and lay the foundation for developing novel insecticidal lead compounds targeting this channel.

The molecular docking results provide computational support for the predicted mechanism of action of Ed-79 [41,42]. The peptide forms an extensive hydrogen-bond network with pore-region residues Pro475, Asn482, Tyr483, and Ser479 of the Shaker Kv channel [43,44], involving multiple polar residues from Ed-79, including Arg3, Ser10, Ala13, Arg21, Lys26, and Thr27. Among these, Pro475 and Asn482 each participate in three hydrogen bonds, suggesting that they may play important roles in Ed-79 recognition. This binding mode is reminiscent of that of classical channel blockers such as Tityustoxin-Kα [45,46], which inhibit ion currents by occluding the outer vestibule above the selectivity filter of potassium channels [47]. Although Ed-79 shares low sequence homology with classical anemone toxins such as ShK, its predicted binding mode is similar to that of these toxins, involving binding to the extracellular pore region and thereby causing steric blockade.

Sequence alignment in the Introduction further revealed that Ed-79 shares a conserved cysteine framework with six previously reported insecticidal or potassium channel-blocking peptides (Figure 1), reinforcing its potential as a novel insecticidal lead compound. While some of these homologs have been shown to be effective against agricultural pests such as cotton bollworm and aphids [48], whether Ed-79 also exhibits activity against these pests remains to be determined, and future studies are needed to experimentally evaluate its insecticidal spectrum.

Several limitations of the present study should be acknowledged. First, the insecticidal activity was assessed by injection, which bypasses the gut barrier and first-pass metabolism, and thus may not fully reflect the efficacy of Ed-79 under field conditions (e.g., via oral administration). Future work should evaluate its oral bioavailability through feeding assays with artificial diets or via transgenic plant expression systems. Second, although molecular docking provides valuable mechanistic insights, it remains a computational prediction and does not constitute experimental evidence that Ed-79 actually inhibits Shaker Kv channel currents. No patch-clamp or two-electrode voltage-clamp recordings were performed in this study to test whether Ed-79 blocks the channel. Patch-clamp recordings [49] in heterologous expression systems, such as Xenopus oocytes or HEK293 cells [50,51], will be essential to definitively establish the functional blockade of the channel by Ed-79.

In summary, this study successfully identified and characterized the sea anemone-derived peptide Ed-79, detailing its chemical synthesis, oxidative folding, insecticidal activity, and predicted molecular binding mode with the Shaker Kv channel. Our findings not only expand our understanding of sea anemone venoms as underexplored resources for insecticide discovery but also provide a promising lead molecule for the development of next-generation, environmentally friendly biopesticides targeting insect potassium channels [52,53].

## 4. Materials and Methods

### 4.1. Screening and Sequence Analysis of the Anemone Peptide Ed-79

The target peptide Ed-79 analyzed in this study was derived from the anemone transcriptome and venom protein database previously established by our research group. A multi-step screening strategy was employed: (1) sequences containing the conserved ShK-family cysteine framework were extracted; (2) sequences with a predicted signal peptide and a mature peptide region of 30–50 amino acids were retained; (3) candidates containing the conserved Lys/Tyr functional dyad typical of Kv1 channel-blocking peptides were selected; and the remaining candidates were subjected to BLASTp (https://blast.ncbi.nlm.nih.gov/Blast.cgi, accessed on 16 September 2026) searches against the NCBI non-redundant protein (nr) and UniProtKB databases. A novel 37-residue peptide containing six cysteine residues was identified and designated Ed-79.

To preliminarily assess the insecticidal potential of Ed-79, the BLASTp and TBLASTn search results were further analyzed, with an E-value threshold of 1 × 10^−5^. The search identified six homologous peptides that have been reported to block Shaker Kv1 potassium channels or exhibit insecticidal activity. Multiple sequence alignments of these peptides together with Ed-79 were conducted using Jalview (version 2.11.5.2) with default parameters (BLOSUM62 scoring matrix and ClustalW algorithm version 2.1 progressive alignment algorithm). By comparing the conserved cysteine backbone and the distribution patterns of key amino acid residues, we found that Ed-79 retains the core structural features and functional sites of known Shaker Kv channel-blocking peptides.

To further analyze the spatial conformation and potential functional interfaces of Ed-79 and its homologs, three-dimensional structure prediction was performed using AlphaFold3 [54,55,56]. For each peptide, five independent models were generated based on multiple sequence alignment-derived evolutionary information. The model with the highest confidence (average pLDDT > 90) was selected for subsequent analysis. All modeled structures were visualized and superimposed using PyMOL (version 2.5).

### 4.2. Synthesis of Linear Peptides

The linear peptide was synthesized via solid-phase peptide synthesis (SPPS) using a CEM Liberty Blue 2.0 automated microwave peptide synthesizer (CEM Corporation, Matthews, NC, USA) [57,58]. Cysteine residues were introduced either as free cysteine or with thiol-protecting groups, specifically S-acetamidomethyl (Acm) and tert-butyl (tBu); all other amino acids were standard Fmoc-protected derivatives, and Rink Amide-AM resin was used as the solid support. Ed-79 belongs to the ShK domain family and contains a conserved cysteine motif (C-C-C-C-X_3_-C-X_2_-C) with specific disulfide connectivity (C1–C6, C2–C4, C3–C5). To achieve the correct disulfide pairing, orthogonal protecting groups were selected: Acm for Cys2 and Cys4, and tBu for Cys3 and Cys5.

After dissolving the amino acids in dimethylformamide (DMF), the synthesizer assembled the peptide sequentially from the C-terminus to the N-terminus according to the programmed sequence. The peptide resin was washed with dichloromethane and dried. Cleavage was performed in a 20 mg/mL cleavage cocktail (92.5% TFA, 2.5% TIPS, 2.5% DODT, and 2.5% H_2_O) at 40 °C for 40 min. After filtration, three volumes of ice-cold diethyl ether (pre-cooled with dry ice) were added, and the mixture was centrifuged and washed (4 °C, 12,000 rpm, 5 min) three times. The resulting precipitate constituted the crude linear peptide. Following solvent evaporation under a fume hood, the crude peptide was dissolved in 20% aqueous acetonitrile, lyophilized, and stored for further use.

### 4.3. Analysis, Purification, and Identification of Linear Peptides

The crude linear peptide was purified by reverse-phase preparative HPLC (RP-HPLC) on an Agilent Pursuit XRs 5 C18 column (21.2 × 250 mm) using a P3500 preparative system (Elite (Dalian) Analytical Instrument Co., Ltd., Dalian, China). Mobile phase A was water containing 0.1% TFA, and mobile phase B was acetonitrile containing 0.1% TFA. Elution was carried out with a linear gradient of 5–50% mobile phase B over 45 min at a flow rate of 5 mL/min, with UV detection at 220 nm. This purification step yielded a crude peptide purity exceeding 75%, as determined by analytical RP-HPLC (target peak area/total peak area). Analytical RP-HPLC was performed on a Waters XBridge^®^ peptide BEH C18 (4.6 × 250 mm; Waters Corporation, Milford, MA, USA) using the same gradient at a flow rate of 1 mL/min, with UV detection at 220 nm. Fractions in which the target peptide peak accounted for ≥95% of the total peak area were considered pure. Only these pure fractions were pooled, lyophilized, and stored for subsequent experiments.

### 4.4. Oxidative Folding of Linear Peptides

The lyophilized linear peptide was dissolved in methanol–water (1:1, *v*/*v*) at 10 mg/mL, and then an equal volume of acetic acid was added to obtain a final solution containing 50% methanol and 50% acetic acid. Under stirring, a 10 mg/mL iodine solution in methanol was added dropwise until a persistent pale yellow color was observed. The mixture was stirred at room temperature for 3 min to allow dehydrogenation of the two free cysteines, forming the first disulfide bond. An aliquot was withdrawn, quenched with L-ascorbic acid (until colorless), and analyzed by RP-HPLC and LC-MS to confirm that only the first pair of cysteines was oxidized while the Acm-protected cysteines remained intact. For the second oxidation step, the intermediate containing one disulfide bond was treated with iodine in 50% aqueous methanol containing HCl for 70 min to remove the two Acm protecting groups and form the second disulfide bond. The resulting intermediate was characterized by RP-HPLC and LC-MS.

For the final oxidation step, the lyophilized product (2 mg/mL) was dissolved in TFA/DMSO/anisole (97.9:2:0.1, *v*/*v*/*v*) and stirred at 37 °C for 45 min. Subsequently, 40 μL/mg of the same TFA/DMSO/anisole solution was added, and the mixture was incubated in a 65 °C water bath for 3 h. Three volumes of ice-cold diethyl ether were added dropwise, and the mixture was centrifuged and washed (4 °C, 12,000 rpm, 5 min) three times. After air-drying, the crude peptide was dissolved in 20% aqueous acetonitrile, purified by preparative RP-HPLC, and analyzed by analytical RP-HPLC and LC-MS. The pure fractions were combined and lyophilized. Because the stepwise oxidative folding strategy uses orthogonal protecting groups, only one specific pair of cysteines is available for oxidation at each step, greatly minimizing the possibility of disulfide-bond isomer formation. Therefore, the connectivity (C1–C6, C2–C4, C3–C5) is predetermined by the protection scheme.

### 4.5. Circular Dichroism Spectroscopy of Peptides

Circular dichroism (CD) spectroscopy is a primary method for studying protein secondary structure and was used to characterize the conformational changes of Ed-79. The lyophilized peptide powder was dissolved in a buffer solution (50% acetonitrile/50% water) to a final concentration of 0.2 mg/mL. CD spectra were recorded from 190 to 400 nm using a MOS-500 CD (Bio-Logic Science Instruments, Seyssinet-Pariset, France) spectrometer in a quartz cuvette with a 0.2 cm path length, using a response time of 1.0 s, a bandwidth of 1.0 nm, and a step size of 1.0 nm. Background spectra of the buffer alone were subtracted from each sample spectrum. For secondary structure analysis, the CD data were converted to mean residue ellipticity (MRE), [θ] (degrees·cm^2^·dmol^−1^·residue^−1^). Helix content was calculated using the CDSSTR algorithm with a reference protein database, accessible via the DICHROWEB website (http://dichroweb.cryst.bbk.ac.uk/html/home.shtml, accessed on 8 October 2026).

### 4.6. Insecticidal Activity Assay

Fourth- to fifth-instar larvae of *Spodoptera frugiperda* and *Tenebrio molitor* with an average weight of 160 mg were used as test insects and were maintained on a normal diet prior to the experiment. A 0.7% saline solution was prepared as the solvent, and the peptide was diluted to concentrations of 2.5, 5, 10, 15, and 20 μM. Approximately 5 μL of each solution was injected into the lower abdomen of the larvae using a fine-tipped microsyringe. For each concentration, 9–11 larvae were injected, and three independent replicates were performed. Negative control groups received an equal volume of 0.7% saline only, and blank control groups received no treatment. All insects were kept under suitable conditions, and mortality was recorded after 24 h.

Peptide preparation and dose calculation. The fully oxidized Ed-79 peptide (molecular weight 3999.08 Da) was weighed (2 mg) and dissolved in 500 μL of solvent (50% acetonitrile in water, then diluted with 0.7% saline) to obtain a 1 mM (1 nmol/μL) stock solution. The stock solution was further diluted with 0.7% saline to prepare working solutions at final concentrations of 2.5, 5, 10, 15, and 20 μM. For each treatment, 5 μL of the working solution was injected into the lower abdomen of each larva (average body weight 160 mg). The injected dose per unit body weight was calculated as follows:

Dose (mol/g) = concentration (mol/mL) × injection volume (mL)/larval body weight (g)

Mortality (%) was calculated as (number of dead larvae/total number of larvae) × 100.

### 4.7. Molecular Docking

Homology modeling of Ed-79 was performed using AlphaFoldv3.0, and molecular docking with the Shaker Kv channel (PDB ID: 7SIP) was carried out using Discovery Studio 2023 SP1 (BIOVIA, Dassault Systèmes, San Diego, CA, USA) software. The protein structures were preprocessed using the Protein Preparation Wizard module in Discovery Studio 2023 SP1, including hydrogen atom addition and water molecule removal. Rigid docking conformations were scored using the ZDOCK algorithm, and the binding conformations of the protein–peptide complexes were optimized and rescored using RDOCK with an energy-based scoring function, both of which are integrated modules within Discovery Studio 2023 SP1. Final docking results were visualized and analyzed using PyMOL 2.6. Since 7SIP is a ligand-free structure without a co-crystallized peptide ligand, conventional redocking validation was not feasible. The reliability of the docking protocol was supported by the established benchmark performance of ZDOCK/RDOCK on protein–peptide complexes, and the predicted binding mode was cross-validated against the known pore-blocking mechanism of Shaker Kv channel inhibitors.

### 4.8. Statistical Analysis

Data processing and statistical analyses were performed using GraphPad Prism v10.1.2. All data were presented as mean ± SEM and compared using one-way ANOVA.

## 5. Conclusions

This study is the first to systematically report the identification of a novel 37-amino-acid peptide, Ed-79, containing three disulfide bonds, from the transcriptome of the sea anemone *E. diaphana*. Using SPPS and a three-step oxidative folding strategy, we successfully obtained the bioactive peptide with correctly paired disulfide bonds, and CD spectroscopy confirmed an increased β-sheet content after folding. Indoor insecticidal assays demonstrated that Ed-79 exhibited dose-dependent lethal effects against *S. frugiperda* and *T. molitor*, with LD_50_ values of 289.5 and 367.2 pmol/g, respectively. Molecular docking predicted that Ed-79 binds stably to the outer pore region of the Shaker Kv channel through eight hydrogen bonds, with Pro475 and Asn482 potentially serving as key interacting residues. This work provides an important basis for the development of novel green insecticidal lead compounds and expands our understanding of sea anemone venom as a valuable resource for insecticide discovery. However, the channel-blocking activity of Ed-79 remains to be experimentally validated by electrophysiological studies.

## Data Availability

The original contributions presented in this study are included in the article/Supplementary Material. Further inquiries can be directed to the corresponding author(s).

## Supplementary Materials

The following supporting information can be downloaded at: https://www.mdpi.com/article/10.3390/molecules31213822/s1, Table S1. Secondary structure contents of Ed-79 and its oxidative folding intermediates determined by CD spectroscopy.
