# Supplementary material for: A Novel Sea Anemone Peptide Ed-79 Blocks Shaker Kv Channels and Exhibits Insecticidal Activity
Source: Molecules. 2026 Oct 23;31(21):3822. doi: 10.3390/molecules31213822 (PMC13764803; doi:10.3390/molecules31213822)
Supplement: Supplementary file 1 [file molecules-31-03822-s001.zip › molecules-4600475-supplementary.pdf]

Table S1. Secondary structure contents of Ed-79 and its oxidative folding intermediates determined by CD spectroscopy.

Analogue	Helix	Strand	Turns	Unordered	Total
Ed-79	0.404	0.159	0.220	0.315	0.991
Ed-79-1	0.361	0.196	0.201	0.290	1.047
Ed-79-2	0.324	0.220	0.215	0.282	1.041
Ed-79-3	0.343	0.225	0.242	0.335	1.145
